# Anaplastic Thyroid Cancer: Clinical Picture of the Last Two Decades at a Single Oncology Referral Centre and Novel Therapeutic Options

**DOI:** 10.3390/cancers11081188

**Published:** 2019-08-15

**Authors:** Joana Simões-Pereira, Ricardo Capitão, Edward Limbert, Valeriano Leite

**Affiliations:** 1 Serviço de Endocrinologia, Instituto Português de Oncologia de Lisboa, Francisco Gentil, Rua Professor Lima Basto, 1099-023 Lisboa, Portugal; 2 Unidade de Investigação em Patobiologia Molecular, Instituto Português de Oncologia de Lisboa, Francisco Gentil, Rua Professor Lima Basto, 1099-023 Lisboa, Portugal; 3 Nova Medical School | Faculdade de Ciências Médicas da Universidade Nova de Lisboa, Campo Mártires da Pátria, n.º 130, 1169-056 Lisboa, Portugal; 4 Serviço de Endocrinologia, Hospital Egas Moniz, Centro Hospitalar Universitário Lisboa Ocidental, Rua da Junqueira, n.º 126, 1349-019 Lisboa, Portugal

**Keywords:** anaplastic thyroid cancer, survival, tyrosine kinase inhibitors, immune checkpoint inhibitors

## Abstract

Anaplastic thyroid cancer (ATC) is a rare tumour but also one of the most lethal malignancies. Therapeutic modalities have usually been limited, but clinical trials with new drugs are now being implemented. The aims of this study were to analyse the clinical presentation, therapeutic modalities and independent prognostic factors for survival. We also reviewed the most recent literature on novel ATC therapies. We performed a retrospective analysis of 79 patients diagnosed between 2000 and 2018. Variables with impact on survival were identified using the Cox proportional-hazard regression model. At presentation, 6.3% had thyroid-confined disease, 30.4% evidenced extrathyroidal extension and 60.8% were already metastatic. Surgery was feasible in 41.8% and radiotherapy was applied to 35.4%, with those receiving >45 Gy having longer estimated survival (*p* = 0.020). Chemotherapy, either conventional or with tyrosine kinase inhibitors, was performed in 17.7% and 7.6%, respectively. Multimodality therapy with surgery, radiotherapy and chemotherapy/tyrosine kinase inhibitors (TKI) had the greatest impact on disease specific survival (DSS), providing a risk reduction of death of 96.9% (hazard ratio (HR) = 0.031, 0.005–0.210, *p* < 0.001). We concluded that most of these patients join reference centres at advanced stages of disease and multimodality treatment may offer the best chances for prolonging survival.

## 1. Introduction

Anaplastic thyroid cancer (ATC) is a rare tumour but accounts for more than 50% of all deaths attributable to thyroid tumours [1]. Undeniably, ATC is one of the most lethal malignancies in humans, as it portends a median survival of only three to four months and a disease-specific mortality at one year of nearly 100% [2]. ATC’s loco-regional invasiveness may cause compressive symptoms, like dysphagia, dyspnoea, stridor and pain, and makes it usually inoperable. Therefore, this disease must be promptly recognized, in order to be managed in a specialized centre, by a multidisciplinary team with expertise in ATC, including endocrinologists, oncologists, surgeons, radiotherapists and pathologists [3]. 

Given its aggressiveness, the American Joint Committee on Cancer (AJCC) classifies all cases of ATC as stage IV: IVA describes all the tumours that are confined to the gland, IVB represents ATC with gross extrathyroidal extension and in stage IVC the tumour has already spread to distant sites [4]. The American Thyroid Association (ATA) guidelines [4] consider multimodal therapy with surgery, radiotherapy and/or chemotherapy, when feasible, as a reasonable approach in these cases; however, a significant proportion of the patients arrive to the health care centres at an advanced stage of disease and, to most of them, palliative and symptomatic care represent the wisest attitude to take.

Different from well-differentiated thyroid cancers (WDTC), ATC cells do not produce thyroglobulin, so there is no tumour marker for these tumours, and they do not respond to thyroid-stimulating hormone (TSH) suppression; furthermore, ATC cells do not uptake iodine, so radioiodine is ineffective in these patients [3]. Given the rarity of ATC, the studies analysing the impact of different therapeutic approaches are small and usually of a retrospective nature or they only report isolated cases. ATC is generally refractory to standard therapies, which renders these patients excellent candidates for innovative adjuvant therapies, including drugs targeting specific oncogenic mutations, tyrosine kinase inhibitors (TKI) and immune-modulating approaches [3]. 

The purpose of this study was to analyse the clinical presentation, therapeutic modalities and its outcomes, as well as the prognostic factors influencing disease specific survival (DSS), in a homogenous population of ATC followed at a single institution. Furthermore, we also reviewed the most recent literature on novel ATC therapies.

## 2. Materials and Methods

We performed a retrospective analysis of patients’ clinical files with ATC diagnosed between 2000 and 2018 and followed at Instituto Português de Oncologia de Lisboa, Francisco Gentil, Lisboa, Portugal. ATC diagnosis was made from cytological or histological specimens and confirmed by experienced pathologists. Cases in which WDTC represented the main counterpart of the histological piece were excluded.

Patients were identified from the Endocrinology and Pathology database. Data regarding demographic characteristics, clinical presentation, therapeutic approaches and its outcomes, and survival were collected and registered with Microsoft Excel (Microsoft, Redmond, WA, USA).

DSS after ATC diagnosis was analysed using the Kaplan–Meier method and log-rank test. The independent prognostic factors were identified using the Cox proportional-hazard regression model. A *p*-value < 0.05 was considered statistically significant. The statistical analysis was performed with IBM SPSS Version 23. 

This study was approved by the Ethics Committee of Instituto Português de Oncologia de Lisboa Francisco Gentil (project number 1056).

## 3. Results

**Figure 1:**
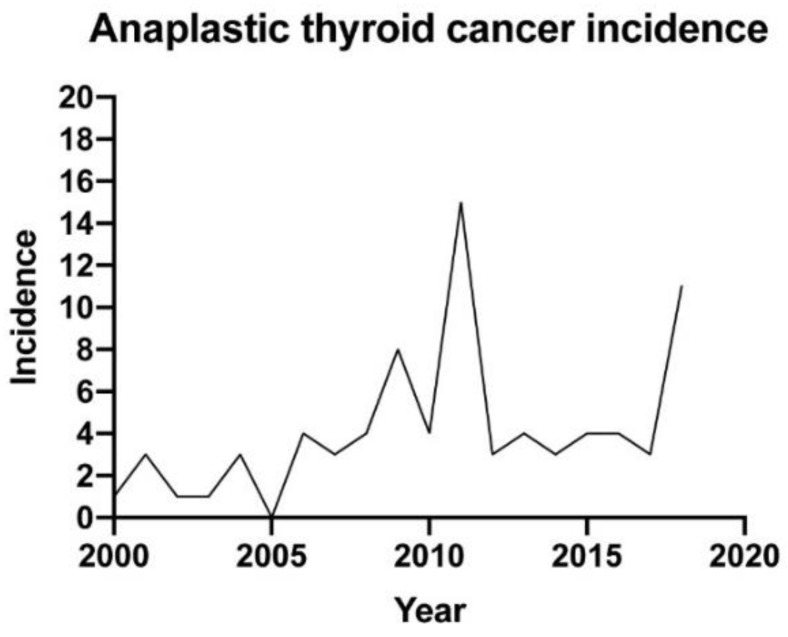
Anaplastic thyroid cancer incidence.

We included 79 patients, 53 (67.1%) of which were women. The median age at diagnosis was 74 (interquartile range (IQR): 15 years). The main clinical characteristics and their impact on median survival are presented in Table 1. The incidence of new ATC diagnosis over the last two decades at our institution is represented in Figure 1.

The majority of patients (86.1%) presented compressive symptoms: 44 (55.7%) with dyspnoea, 32 (40.5%) with dysphagia, 20 (25.3%) with dysphonia and 25 (31.6%) complained of local pain; 31 (39.2%) patients had to be submitted to tracheostomy and 2 (2.5%) to tracheal prosthesis insertion; 8 (10.1%) needed a percutaneous endoscopic gastrostomy. 

Almost half of the patients (37 (46.8%)) had a previous history of multinodular goiter. In the histological specimen, nine (11.4%) patients evidenced a WDTC area; three of these had been followed for a papillary thyroid cancer in the previous years and underwent subsequent dedifferentiation.

At presentation, more than 60% of the patients presented distant disease. The lungs were the most common site of metastases (45 (57.0%)), followed by bones (7 (14.3%)) and the brain (3 (4.0%)); in 39 patients (49%) only one tissue was affected.

**Figure 2:**
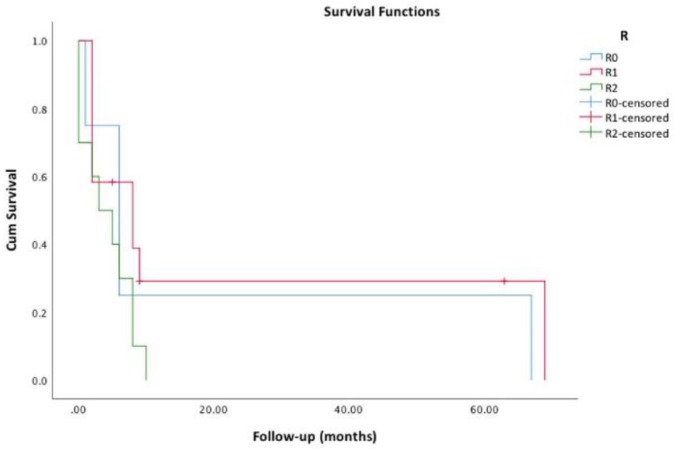
Survival functions regarding surgical margins (R0 vs. R1 vs. R2).

**Figure 3:**
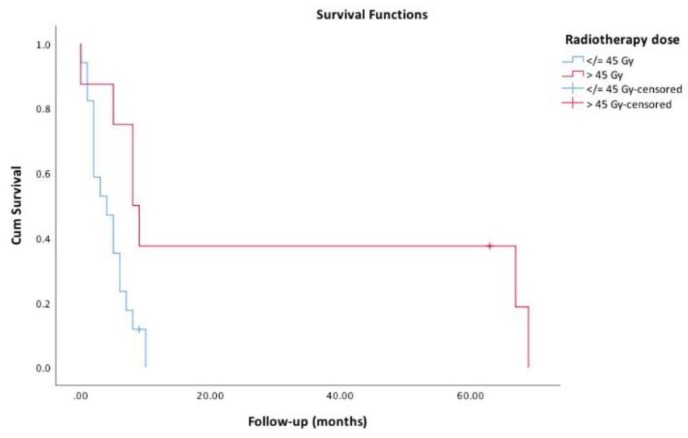
Survival functions regarding radiotherapy dose (≤45 Gy vs. > 45 Gy).

The diagnostic approaches that were offered to our patients are presented in Table 1. Of the 33 (41.8%) patients who were submitted to surgery, resection margins were negative (R0) in 4 (12.5%), microscopic (R1) in 12 (37.5%) and macroscopic (R2) in 10 (31.3%). Surgical margins did not have impact on survival: estimated median survival for R0, R1 and R2 patients was 6.0, 8.0 and 3.0 months (*p* = 0.177), respectively (Figure 2). Radiotherapy was offered to 28 (35.4%) patients; median radiation dose was 28.8 (IQR: 46) Gy. Patients who received more than 45 Gy had longer estimated survival: 8.0 vs. 4.0 months, *p* = 0.020 (Figure 3). Additionally, seven (8.8%) patients were also submitted to radiosensitizing chemotherapy, yet although their estimated survival was higher, it did not reach statistical significance compared to radiotherapy alone (6.0 vs. 4.0 months, *p* = 0.085). Altogether, 14 (17.7%) patients were submitted to chemotherapy; the protocols included platin, taxane and/or anthracycline, as recommended by ATA guidelines [4], but the different regimens did not influence survival (*p* = 0.203). TKIs were offered to six (7.6%) patients: sorafenib to four, lenvatinib to one and sunitinib after progression with sorafenib to another patient.

Median estimate DSS was 2.0 months. However, four (5%) patients lived longer than one year. All of these were submitted to thyroidectomy with a multimodal approach. One patient remains alive, without evidence of disease, after 63 months of follow-up—he was 60 years old when he was submitted to thyroidectomy (surgery was R1), the tumour had well-differentiated areas, and he was also treated with adjuvant radiotherapy (total dose of 66 Gy) and chemotherapy with doxorubicin + docetaxel.

Multivariate analysis is shown in Table 2. Stage IVC, that is, metastatic disease, showed a tendency to influence survival. However, only the therapeutic approaches had a significant impact on overall survival: multimodality therapy with surgery, radiotherapy and chemotherapy/TKI had the greatest impact, providing a risk reduction of 96.9% of death for each month of follow-up, followed by surgery + radiotherapy, surgery + chemotherapy/TKI, surgery alone, and chemotherapy/TKI and/or radiotherapy, when comparing to isolated symptomatic care. 

## 4. Discussion

ATC is an orphan disease that portends a very dismal prognosis. Multimodal approaches that include surgery have the largest impact in improving survival. At our institution, ATC diagnosis has shown a tendency to increase over the years and not to decrease (Figure 1), as observed by other authors [5,6]. We observed that our cases continue to be detected at later stages, since only 6.3% of the patients evidenced thyroid-confined disease at presentation. Accordingly, almost 90% had compressive symptoms, mainly dyspnoea. Furthermore, 60.8% had distant metastases at presentation. This is certainly due to the rapid growth typical of these tumours, but we cannot exclude a referral bias, since our hospital is a tertiary care centre. Furthermore, we also admit the unrecognition of the disease by primary and secondary care because almost half of our cohort had a previous history of a long-standing multinodular goiter, which represents a well-recognized risk factor for ATC, especially in elderly patients [3]. As verified by other authors [7,8], lungs were the primary sites of metastases (57%). Bone came as the second most common affected tissue (14.3%) which differs from other publications in which intrathoracic lymph nodes were the second most affected distant site. Six patients with IVC stage also showed coexistent well-differentiated areas at histology (data not shown), but we could not assess whether their metastases were from the well-differentiated or from the undifferentiated component. 

In our cohort we identified, by univariate analysis, some characteristics that influenced survival: stage at diagnosis, leukocytosis, method of diagnosis, previous or coexistent WDTC and therapeutic approach. On multivariate analysis, despite the tendency for metastatic disease to influence overall survival, only the therapeutic approach had a strong statistical significance. Compared to isolated symptomatic care, surgery alone provided a risk reduction of death of 71.1%, but when combined with radiotherapy or chemotherapy/TKI this reduction was even greater (89.2% and 84.8%, respectively). The multimodality approach that included surgery plus radiotherapy and chemotherapy/TKI had the most beneficial impact on survival, portending a risk reduction of 96.9%. Interestingly, we verified that the amount of residual disease, either complete (R0 and R1) or incomplete (R2) surgery, did not affect survival. On the other hand, radiation dose of >45 Gy provided a longer survival. Neither the used regimens of chemotherapy nor the TKIs were associated with survival, but the number of patients who were submitted to these therapies was small, which hampered the analysis of their individual impact on prognosis. Different prognostic variables have been suggested in ATC. For instance, Glaser et al. [9] analysed the American National Cancer Database (NCDB) and identified total thyroidectomy and high-dose radiotherapy as independent prognostic factors in a cohort of 3552 patients. Lennon et al. [10] studied 64 Irish patients and recognised, as independent negative factors of survival, the presence of distance metastases and palliative care. In a smaller cohort, Machens et al. [11] reported that only nodal status influenced survival. Other authors [12] verified that age ≥70 years, presence of acute symptoms and leukocytosis at initial diagnosis, pT4b tumour, largest tumour dimension >5 cm and distant metastases, were all independent predictive factors of poorer survival. A multicentre German study that recently analysed a cohort of 100 ATC patients [13] reported similar demographic and clinicopathological characteristics to our patients; they found that radical surgery with sequential or simultaneous chemotherapy and radiotherapy was associated with survival in IVC patients (HR = 0.1, 0.03–0.31, *p* < 0.001). However, they noticed that surgery combined with either chemo or radiotherapy alone had no statistically significant benefit compared with surgery alone in their series of IVC patients. Also, in a cohort of 100 patients from Japan, Akaishi and colleagues [14] identified age ≥70 years, leukocytosis, extrathyroid invasion, distant metastases at diagnosis and, with greater impact, complete resection and radiotherapy ≥40 Gy, as independent prognostic factors, which is similar to our results. Haymart et al. [15] analysed the overall survival of 2742 ATC patients diagnosed between 1998 and 2008 and concluded that longer survival was associated with a more intensive and multimodal therapy. A study of 95 patients followed at Memorial Sloan-Kettering Cancer Centre [16] verified that in patients with locoregional disease, multimodality treatment with gross total surgical resection and postoperative radiotherapy with or without chemotherapy provided the best local control and had the greatest beneficial impact on DSS.

According to our results and to other reports [14,17,18,19], including a research by Pezzi and colleagues [17], that enrolled 1288 patients from the American NCDB, radiotherapy with cumulative doses of >40/45 Gy compared to lower dose regimens provided longer survival. 

Only 17% of our patients were submitted to chemotherapy, making it difficult to draw any conclusion regarding the prognostic significance of this form of therapy. In general, chemotherapy response rates are very low in ATC (15–25%), bringing only a short period of benefit [20]. Ain et al. [21] reported a phase 2 clinical trial of a 96-h infusion of paclitaxel with the most satisfactory results, showing a total response rate of 53%. Chemotherapy in the neoadjuvant setting has only been evaluated in small studies (reviewed in [22]). One of these studies [23] investigated the effectiveness of weekly paclitaxel in stage IVB (*n* = 9) and observed a response rate of 33%.

### Precision Medicine in ATC: Target and Immune Modulation Therapies

As also observed in our cohort, ATC has been identified in coexistence with well-differentiated areas, suggesting that these tumours can develop from pre-existing WDTC cases. On the other hand, it has also been hypothesised that these tumours may arise de novo. It has been postulated that the presence of mutated *RAS* and *BRAF* in follicular thyroid cancer (FTC) and papillary thyroid cancer (PTC), respectively, in association with later acquired alterations in *TP53* and mTOR pathway, might be responsible for this process of dedifferentiation [2,24,25]. Our group has studied the mutational profiles of genes involved in the most deregulated cellular processes and molecular pathways in poorly-differentiated thyroid cancer (PDTC) and ATC [26]. We found that most mutations were present in *TP53* and *RAS* genes. Mutations in *CDKs*, *PIK3CA* and *PTEN* were also present. In a recent study, Kunstman et al. [27] performed whole-exome sequencing in 22 ATC and 4 cell lines, finding the majority of variants clustered in the MAPK, ErbB and RAS signalling pathways. Somatic mutations in established thyroid cancer related genes (e.g., *BRAF*, *TP53*, *RAS*, *CDKI*, *PIK3CA*) were detected in 64% of those tumours. In addition, mutations in genes not previously associated with thyroid tumourigenesis were also observed (e.g., *mTOR*, *NF1*, *NF2*, *MLH1*, *MLH3*, *MSH5* and *MSH6*). Landa et al. [28] also performed next-generation sequencing (NGS) of 341 cancer genes from 117 patient-derived PDTC and ATC and analysed the transcriptome of a representative subset of 37 tumours and found a high prevalence of *TP53*, *TERT* promoter, PI3K/AKT/mTOR pathway effectors, SWI/SNF subunits, and histone methyltransferases, beyond the *BRAF* and *RAS* mutations, which were the predominant drivers. The knowledge of the genomic landscape of ATC is becoming very important in clinical practice given that techniques like NGS or liquid biopsies enable a complete somatic analysis in only a few days. Different TKIs that target the abovementioned pathways have been developed and studied in ATC patients.. The mechanisms of action underlying the therapeutic effects of these drugs have been previously reviewed by our group [29] (see Table 3).

Therapy with lenvatinib and sorafenib were approved in WDTC by the Food and Drug Administration (FDA) and by the European Medicines Agency (EMA); however, these TKIs are not currently authorized in ATC by these regulatory agencies. However, lenvatinib was approved in Japan for ATC treatment after a phase II trial (NCT01728623) [30]. In the last year, the FDA approved a *BRAF* inhibitor (dabrafenib) in combination with a *MEK* inhibitor (trametinib) for *BRAF*-mutated ATC, based on a phase II trial (NCT02034110) [31]. The rationale behind this association is that the *MEK* inhibition prevents the rebound activation of MEK and ERK pathway after *BRAF* inhibition; this combination results in enhancement of BRAF-MEK-ERK pathway inhibition and anti-tumour activity [32]. Table 3 shows the drugs that have been approved and studied in ATC patients.

Tumour-infiltrating immune cells are present in most solid tumours and can affect the response to therapy and thus clinical outcome. Advances in immune-mediated therapies have evidenced the importance of expanding our understanding of thyroid cancer progression from intrinsic oncogenic pathways to the whole tumour microenvironment. Recently, some studies analysed the presence of certain immune cells, like tumour-associated macrophages, which seem to be prognostic factors in ATC [33]. Chintakuntlawar et al. [34] also investigated the prognostic value of the immune checkpoints programmed death-1 (PD-1) and its ligand (PD-L1) in these tumours and verified that PD-1 and PD-L1 are both highly expressed in ATC and are associated with shorter progression-free survival and overall survival in multimodality-treated patients. Different authors have reported the outcomes of distinct immune checkpoint inhibitors on ATC treatment (see Table 4), including pembrolizumab, a monoclonal antibody against PD-1 receptor approved by the FDA in different types of cancer. For instance, Iyer et al. [35] described a cohort of 12 patients with ATC who were treated with pembrolizumab in combination with TKI at the time of progression on TKI; they observed that the addition of pembrolizumab enabled a median overall survival and progression-free survival of 6.93 and 2.96 months, respectively. One year earlier, Kollipara et al. [36] had reported a clinical case of an ATC patient that showed complete remission after treatment with vemurafenib plus nivolumab (therapeutic choices guided by NGS results).

The studies analysing the effects of TKI and immune checkpoint inhibitors either isolated or in association are presented in Table 4. In Table 5 we show the clinical trials with these therapies that are currently recruiting or ongoing (www.clinicaltrials.gov; www.clinicaltrialsregister.eu; accessed on 18 May 2019).

## 5. Conclusions

The present study reflects the largest experience with ATC in the country and is one of the largest reported in Europe. One limitation is its retrospective nature, which makes it difficult to retrieve data not stated in clinical files, but this problem is similar to the majority of the studies reported with this type of cancer.

The major issue about ATC, apart from the lack of effective therapeutic options, is that the great majority of patients join the reference centres at an advanced stage of disease. Thus, it is urgent to improve and speed up the recognition and referral of these aggressive cases to tertiary or oncological centres so that they can be promptly managed by a multidisciplinary team that offers a multimodality treatment, which is often complex and needs to be held at an institution with expertise in ATC. Recently, Cabanillas et al. [53] implemented at their centre a new process flow for patients with ATC, allowing their immediate schedule, which resulted in a 98% decrease in referral to disposition time and led to a significant increase in ATC referrals to their institution.

In conclusion, ATC management is very challenging, and prospective trials are needed, with clinical criteria that may be adjusted to the real ATC patients, who are usually old and with an advanced stage of disease. Multimodality approach with surgery, radiotherapy and chemotherapy, either conventional or with the new agents, may offer the best chances for survival enhancement.

## Figures and Tables

**Table 1:** Clinicopathological characteristics.

Characteristics	No. (%)	Median Survival, mo	*p*-Value, Log-Rank Test
Age			
≤70 years	36 (45.6%)	2.0	0.514
>70 years	43 (54.4%)	2.0	
Sex			
Female	53 (67.1%)	2.0	0.484
Male	26 (32.9%)	3.0	
Tumour dimensions			
≤60 mm	32 (40.5%)	2.0	0.511
>60 mm	34 (43%)	2.0	
NA	13 (16.5%)		
Stage at diagnosis			
IVA	5 (6.3%)	9.0	
IVB	24 (30.4%)	2.0	0.031
IVC	48 (60.8%)	2.0	
NA	2 (2.5%)		
Leukocytosis			
<10,000/mm^3^	12 (15.2%)	1.0	
10,000–20,000/mm^3^	44 (55.7%)	2.5	0.003
20,000–30,000/mm^3^	12 (15.2%)	1.0	
>30,000/mm^3^	4 (5.1%)	1.0	
NA	7 (8.9%)		
Diagnosis			
Cytology	36 (45.6%)	1.0	0.001
Histology	43 (54.4%)	2.0	
Compressive symptoms			
Yes	68 (86.1%)	1.0	0.451
No	9 (11.4%)	2.0	
NA	9 (11.4%)		
Previous or coexistent WDTC			
Yes	9 (11.4%)	5.0	0.004
No	63 (79.7%)	2.0	
NA	7 (8.9%)		
Therapeutic approach			
Only symptomatic	31 (39.2%)	<1.0	
Only surgery	11 (13.9%)	2.0	
Only RT	11 (13.9%)	2.5	
Only CT	2 (2.5%)	0.5	<0.001
Only TKI	3 (3.8%)	3.0	
S + RT + CT	6 (7.6%)	38.5	
S + RT	9 (11.4%)	5.0	
S + CT	4 (5.0%)	6.0	
S + RT + CT + TKI	2 (2.5%)	8.0	
S + TKI	1 (1.3%)	9.0	

NA, not available; CT, chemotherapy; RT, radiotherapy; S, surgery; TKI, tyrosine kinase inhibitors; WDTC, well-differentiated thyroid cancer.

**Table 2:** Multivariate analysis.

Variables	Exp(B)	95% CI	*p*-Value
Age	1.005	0.974–1.036	0.765
Stage IVB ^1^	3.098	0.865–10.838	0.077
Stage IVC ^1^	3.327	1.001–11.055	0.050
Leukocytosis ^2^	0.686	0.325–1.449	0.324
Histological diagnosis ^3^	1.265	0.746–3.561	0.221
Previous or concomitant WDTC ^4^	0.719	0.351–4.556	0.719
Only surgery ^5^	0.289	0.101–0.828	0.021
Only chemo/TKI and/or RT ^5^	0.423	0.199–0.900	0.026
Surgery + RT ^5^	0.108	0.034–0.341	<0.001
Surgery + CT/TKI ^5^	0.152	0.041–0.568	0.005
Surgery + RT + CT/TKI ^5^	0.031	0.005–0.210	<0.001

^1^ Reference: stage IVA; ^2^ reference: without leukocytosis; ^3^ reference: cytological diagnosis; ^4^ reference: without WDTC areas; ^5^ reference: only symptomatic therapy. CT, chemotherapy; RT, radiotherapy; S, surgery; TKI, tyrosine kinase inhibitors; WDTC, well-differentiated thyroid cancer.

**Table 3:** Tyrosine kinase and immune checkpoint inhibitors studied in ATC.

TKI or ICI	Targeted Alteration
Recently approved drugs in ATC	
Dabrafenib + trametinib (150 mg twice daily + 2 mg once daily)	BRAF + MEK
Lenvatinib [24 mg daily]	VEGFR, FGFR, PDGFR-α, RET, c-kit, KIF5B-RET, CCDC6-RET, NcoA4-RET rearrangement
Drugs studied in ATC	
Pembrolizumab	PD-1
Nivolumab	PD-1
Spartalizumab	PD-1
Durvalumab	PD-L1
Tremelimumab	CTLA-4
Sorafenib	VEGFR, PDGFR-β, c-kit, RAF, RET, FLT3
Sunitinib	VEGFR, PDGFR, RET, c-kit, FLT3
Vemurafenib	BRAF
Crizotinib	ALK, MET, ROS1
Everolimus	mTOR, PI3K
Pazopanib	VEGFR, FGFR, PDGFR, c-kit
Imatinib	Bcr-Abl, PDGFR, c-kit
Gefitinib	EGFR

ATC, anaplastic thyroid cancer; TKI, tyrosine kinase inhibitors; ICI, immune checkpoint inhibitors.

**Table 4:** Reported results of tyrosine kinase and immune checkpoint inhibitors in ATC.

Authors	Year	TKI or ICI	No. of ATC Patients	Response	Median OS and PFS since TKI/IMT
Sherman et al. [37]	2019	Durvalumab + tremelimumab (+SBRT)	12	ORR: 0 (0%) SD: 1 (8%)	OS: 14.5 weeks
Harris et al. [38]	2019	Everolimus	5	PR: 1 (20%), SD: 2 (40%); PD: 1 (20%)	OS: 7.4 mo
Iyer et al. [20]	2018	Dabrafenib + trametinib Lenvatinib	6 10	PR: 3 (50%); SD: 2(33%) PR: 3 (30%); SD: 4 (40%)	OS: 9.3 mo; PFS: 5.2 mo OS: 3.9 mo; PFS: 2.6 mo
Iyer et al. [35]	2018	Pembrolizumab (added to TKI)	12	PR: 5 (42%); SD: 4 (33%); PD: 3 (25%)	OS: 6.94 mo PFS: 2.96 mo
Wirth et al. [39]	2018	Spartalizumab	30	ORR: 5–6 (17–20%), depending on the criteria	
Subbiah et al. [31]	2017	Dabrafenib † trametinib	16	ORR: 69%	
Tahara et al. [30]	2017	Lenvatinib	17	PR: 4 (24%); SD: 12 (71%); PD: 1 (6%)	OS: 20.6 mo PFS: 7.4 mo
Ito et al. [40]	2017	Sorafenib	10	CR: 0 (0%); PR: 0 (0%); SD: 4 (40%)	OS: 5 mo PFS: 2.8 mo
Ravaud et al. [41]	2017	Sunitinib	4		OS: 5.7 mo PFS: 9.8 mo (2 pts)
Iniguez-Ariza [42]	2017	Lenvatinib	3	PR: 0 (0%); SD: 1 (33%)	OS: 2–7 mo
Kollipara et al. [36]	2017	Vemurafenib † nivolumab	1	CR	
Hyman et al. [43]	2016	Vemurafenib	7	CR: 1 (14%); PR: 1 (14%); SD: 0 (0%); PD: 4 (57%)	
Godbert et al. [44]	2015	Crizotinib	1	Response >90%	
Marten et al. [45]	2015	Vemurafenib	1	PD after 2 mo	
Wagle et al. [46]	2014	Everolimus	1		18 mo
Lim et al. [47]	2013	Everolimus	6	PR: 1 (17%)	
Savvides et al. [48]	2013	Sorafenib	20	PR: 2 (10%); SD: 5 (25%)	OS: 3.9 mo PFS: 1.9 mo
Rosove et al. [49]	2013	Vemurafenib	1	PR	
Bible et al. [50]	2012	Pazopanib	16	PD: 16 (100%)	OS: 111 days PFS: 62 days
Ha et al. [51]	2010	Imatinib	4	PR: 2 (25%); SD: 4 (50%)	6 mo-OS: 46% 6 mo-PFS: 27%
Pennell et al. [52]	2008	Gefitinib	5	PR: 0 (0%)	

CR, complete response; ICI, immune checkpoint inhibitors; mo, months; ORR, overall response ratio; OS, overall survival; PD, progressive disease; PFS, progression-free survival; PR, partial response; SD, stable disease; SBRT, stereotactic body radiotherapy; TKI, tyrosine kinase inhibitors.

**Table 5:** Currently recruiting and ongoing clinical trials of tyrosine kinase and immune checkpoint inhibitors in ATC.

Currently Recruiting				
Study title	Phase	Drug(s)	Year of start	Estimated year of completion
Trametinib in combination with paclitaxel in the treatment of ATC *NCT03085056*	I	Trametinib Paclitaxel	2017	2020
A phase II study of MLN0128 in metastatic ATC *NCT02244463*	II	MLN0128	2014	2022
Nexavar for neoadjuvant treatment of ATC *NCT03565536*	II	Sorafenib	2018	2019
Pembrolizumab in anaplastic/undifferentiated thyroid cancer *NCT02688608*	II	Pembrolizumab	2016	2020
Ceritinib in mutation and oncogene directed therapy in thyroid cancer *NCT02289144*	II	Ceritinib	2014	2021
Nivolumab plus ipilimumab in thyroid cancer *NCT03246958*	II	Nivolumab Ipilimumab	2017	2025
Atezolizumab with chemotherapy in treating patients with anaplastic or poorly differentiated thyroid cancer *NCT03181100*	II	Atezolizumab Bevacizumab Cobimetinib Nab-paclitaxel Paclitaxel Vemurafenib	2017	2023
Ongoing				
Phase II study assessing the efficacy and safety of lenvatinib for ATC *NCT02726503*	II	Lenvatinib	2016	2020
Immunotherapy and stereotactic body radiotherapy (SBRT) for metastatic ATC *NCT03122496*	I	Durvalumab Tremelimumab SBRT	2017	2020
Pembrolizumab, chemotherapy and radiation therapy with or without surgery in treating patients with ATC *NCT03211117*	II	Docetaxel Doxorrubicin hydrochloride IMRT Pembrolizumab	2017	2019
Phase I/II study of PDR001 in patients with advanced malignancies *NCT02404441*	I II	PDR001	2015	2020
Intensity-modulated radiation therapy and paclitaxel with or without pazopanib hydrochloride in treating patients with anaplastic thyroid cancer *NCT01236547*	II	IMRT Paclitaxel Pazopanib hydrochloride	2010	2019
Pazopanib hydrochloride in treating patients with advanced thyroid cancer *NCT00625846*	II	Pazopanib hydrochloride	2008	
Treatment with recombinant human Interleukin 1 receptor antagonist (Anakinra) in patients with anaplastic thyroid cancer: a proof of concept study *EudraCT: 2017-003028-59*	IV	Interleukin 1 receptor antagonist	2018	
A phase II study to investigate the efficacy of RAD001 (Afinitor ^®^, everolimus) in patients with irresectable recurrent or metastatic differentiated, undifferentiated (anaplastic) and medullary thyroid carcinoma *EudraCT: 2009-016669-27*	II	Everolimus	2010	
An open-label phase 2 multi-cohort trial of nivolumab in advanced or metastatic malignancies *EudraCT: 2016-000461-23*	II	nivolumab	2017	
